# A comprehensive study of the rare diseases and conditions targeted by orphan drug designations and approvals over the forty years of the Orphan Drug Act

**DOI:** 10.1186/s13023-023-02790-7

**Published:** 2023-06-23

**Authors:** Lewis J. Fermaglich, Kathleen L. Miller

**Affiliations:** Office of Orphan Products Development, Office of the Commissioner, US Food and Drug Administration, 10903 New Hampshire Ave, Silver Spring, MD 20993 USA

## Abstract

**Background:**

Rare diseases affect more than 30 million Americans. The passage of the Orphan Drug Act (ODA) in the United States in 1983 represented a launching point for a rare disease drug development revolution for these patients. Financial incentives provided by the ODA through its Orphan Drug Designation Program, in addition to remarkable scientific advances over the past 40 years, have led to hundreds of drug approvals for rare diseases. Our research examines the rare diseases that have been targeted by orphan drug designations and subsequent approvals since the law was enacted.

**Methods:**

Using an internal FDA database, we classified and analyzed all orphan drug designations and approvals from 1983 to 2022 by disease and therapeutic area.

**Results:**

Over the 40 years of the ODA, 6,340 orphan drug designations were granted, representing drug development for 1,079 rare diseases. Additionally, 882 of those designations resulted in at least one FDA approval for use in 392 rare diseases. Much of this development has been concentrated in oncology as seven of the top ten most designated and approved diseases were rare cancers.

**Conclusions:**

Researchers have estimated that there may be 7000–10,000 rare diseases that have been identified and described. Based on our study, we can conclude that around 5% of rare diseases have an FDA-approved drug and up to 15% of rare diseases have at least one drug that has been developed and shown promise in their treatment, diagnosis or prevention. Funding of basic and translational science for rare disease drug development should continue in order to bring therapies to the millions of affected patients who remain without treatment options.

## Background

The Orphan Drug Act (ODA) was signed into law in the United States on January 4th, 1983 [1]. Drug development for rare diseases can be commercially risky, and prior to this legislation, only approximately two drugs per year had been approved by the US Food and Drug Administration (FDA) for rare diseases [2]. Now, four decades later, hundreds of “orphan” drugs have been approved for use in the 7000–10,000 diseases and conditions that are considered rare.[3].

In addition to establishing the Orphan Products Grants Program, the ODA created the Orphan Drug Designation Program, which was designed to provide financial incentives for companies developing drugs and biologics for rare diseases and conditions [4]. These incentives currently include: a 25% tax credit on applicable research and development costs; waived FDA user fees (the fee that companies pay to the FDA to offset the cost of application review); and the potential for seven years of marketing exclusivity for an approved orphan-designated indication [4].

To receive orphan drug designation, sponsors must submit a request to the FDA’s Office of Orphan Products Development (OOPD) and meet two principal designation criteria. First, they must demonstrate that the drug or biologic would be used to treat, prevent, or diagnose a rare disease or condition–statutorily defined as affecting fewer than 200,000 patients in the US—and second, provide preclinical or clinical data that establishes a scientific rationale that the drug may be effective in the intended rare disease or condition. Requests for orphan drug designation can be submitted at any time, from early-stage drug development to late-phase clinical trials, prior to the submission of a marketing application [5].

It appears that the financial incentives provided by the ODA, coupled with basic science and translational innovations, have led to significant advances in the treatment of rare diseases. Some of these advances have come from sequencing the human genome which has led to both the enhanced ability to identify rare diseases with the rise of genomic medicine, and has translated into the creation of gene-based and other molecularly targeted therapies [6]. Others have come from advances in the understanding of the progression and variety of clinical manifestations of rare diseases through natural history studies, patient registries, and big data analytics, in turn leading to innovative therapies [7].

These breakthroughs have contributed to a surge of interest in rare disease drug development resulting in an increasing number of orphan drug approvals. Orphan approvals (which include new molecule, indication, and formulation approvals) increased from 14 in 2000 to 77 in 2017 [8]. In 2022, drugs that treated rare diseases represented nearly half (49%) of all novel drugs and biologics approved by the FDA [9, 10].

While orphan drug approvals are the obvious desired result for patients, in the current study we also examine orphan drug designations as a proxy measure of research activity and the pipeline of promising drug candidates for rare diseases. Additionally, we move beyond the traditional classification scheme of therapeutic areas to focus directly on individual rare diseases and conditions that have benefited most from these scientific advances. By analyzing the individual rare diseases receiving designation and subsequent FDA-approval, we are able to craft a more nuanced view of the rare disease medical product development landscape.

## Data and methods

We used an internal FDA database to create a dataset that included all orphan drug designations and approvals from 1983 to 2022 along with characteristics of the designations including: the orphan-designated disease or condition, the date of designation, and the approval date (if applicable). (Public access to the designations and approvals can be obtained via a database published online by the FDA [11]).

The orphan-designated disease or condition is typically a longer phrase that is difficult to aggregate across designations. Thus, to effectively analyze the dataset, it was necessary to convert the orphan-designated disease “phrase” into a simplified, uniform disease “term.” For example, an orphan-designated disease phrase of “maintenance treatment of patients with deficiencies in enzymes of the urea cycle,” was transformed into the disease term “urea cycle disorder.”

To confirm that designated disease terms recognized discrete diseases or conditions, we utilized a standardized disease terminology designed for optimal data integration and harmonization to align disease naming across multiple sources (“Mondo”; [12]). All simplified designated disease terms were assigned a discrete disease label via the Mondo Disease Ontology based on the most granular, lowest hierarchical information as allowed by the original orphan-designated disease phrase.

While the transformation of most designations into simplified disease labels was relatively straightforward, there were some special cases. Designations for conditions that were complications of an underlying disease, side effects of treatments for an underlying disease, or opportunistic diseases primarily associated with an underlying disease were classified as the underlying disease or condition for their term. For example, “treatment of pulmonary fungal infections in patients with cystic fibrosis,” was distilled into “cystic fibrosis.” We chose to use this methodology to keep the analysis patient-centered. Our rationale is that patients with, for example, cystic fibrosis, care not only about the development of drugs to treat their underlying condition, but also the comorbidities that arise because of it, such as opportunistic infections.

Four percent of orphan drug designations could not be classified into a discrete disease label via the Mondo ontology. The majority of these were designations related to or following specific procedures (e.g., surgical or transplant-related complications). In these cases, we aggregated disease terms according to the described related procedure or condition.

Finally, we distributed each discrete disease label into a broader category of “therapeutic area” that could be aggregated across designations. Although many of the designated diseases could be characterized as multisystemic, we based therapeutic area classification on the disease type (e.g., oncology, infectious disease) primarily, and then the most affected organ system (e.g., neurology, dermatology) secondarily (e.g., gastric cancer was categorized as “oncology” rather than “gastrointestinal”).

Orphan approvals can encompass multiple types of approvals, including those for novel drugs or biologics, new indications, and new formulations [8]. For our analysis of orphan approvals, we tallied “total approvals”, which is the sum of all three types of approvals. We then broke “total approvals” into both “initial approvals”, which consisted of the first approval falling under one orphan designation, and “subsequent approvals”, which were approvals that occurred after that first approval. These “subsequent approvals” could have been awarded for additional indications (e.g., clinically studied age expansions, additional mutations clinically responsive to the drug, new line of therapy for oncology drugs, etc.) or for new formulations. Because only certain types of designated products ever receive “subsequent approvals”, analyzing “initial approvals” separately allowed for a more accurate, uniform, and relevant assessment of the true frequency of drugs approved for a particular orphan drug-disease pairing. For example, one drug designated for the treatment of multiple myeloma—daratumumab—has seven “total approvals” from 1983 to 2022 for multiple distinct drug combination regimens and prior lines of therapy, but would account for one “initial approval” and six “subsequent approvals” in this study.

## Results

As of December 31, 2022, OOPD had granted 6340 orphan drug designations which represented 1079 diseases.

From 1983 to 2022, there were 1122 total approvals (including new molecule, indication, and formulation approvals) of orphan-designated products. There were 882 initial approvals (first approval falling under one orphan designation) representing 392 rare diseases. Fourteen percent of designations resulted in at least one orphan drug approval.

**Fig. 1:**
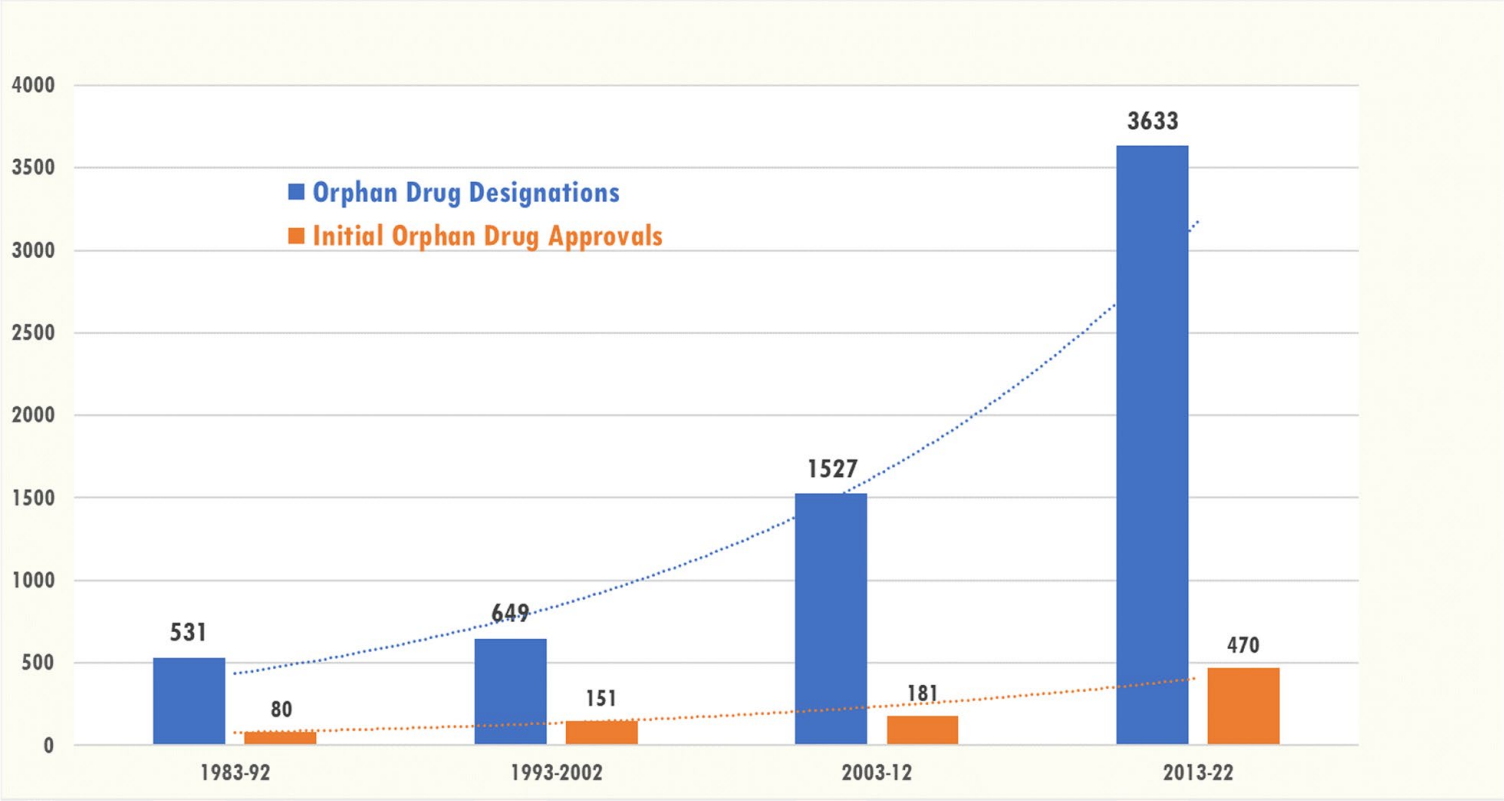
Total orphan drug designations (n = 6340) and initial orphan drug approvals (n = 882) by decade, 1983–2022

The number of both designations and approvals has increased over time: there were nearly seven times as many designations in the most recent decade (2013–2022) as compared to the first decade after the ODA was enacted (1983–1992) and six times the number of initial approvals over the same time period. (Fig. 1).

The median number of designations per disease was two, and the maximum number of designations per disease was 185 (pancreatic cancer). We found that seven diseases had more than 100 associated designations each, 58 diseases had 20 or more designations each, and 442 diseases had only one associated designation. The maximum number of initial approvals was 23 (HIV). Notably, HIV is no longer considered rare by FDA as its current prevalence is greater than 200,000 in the US.

**Fig. 2:**
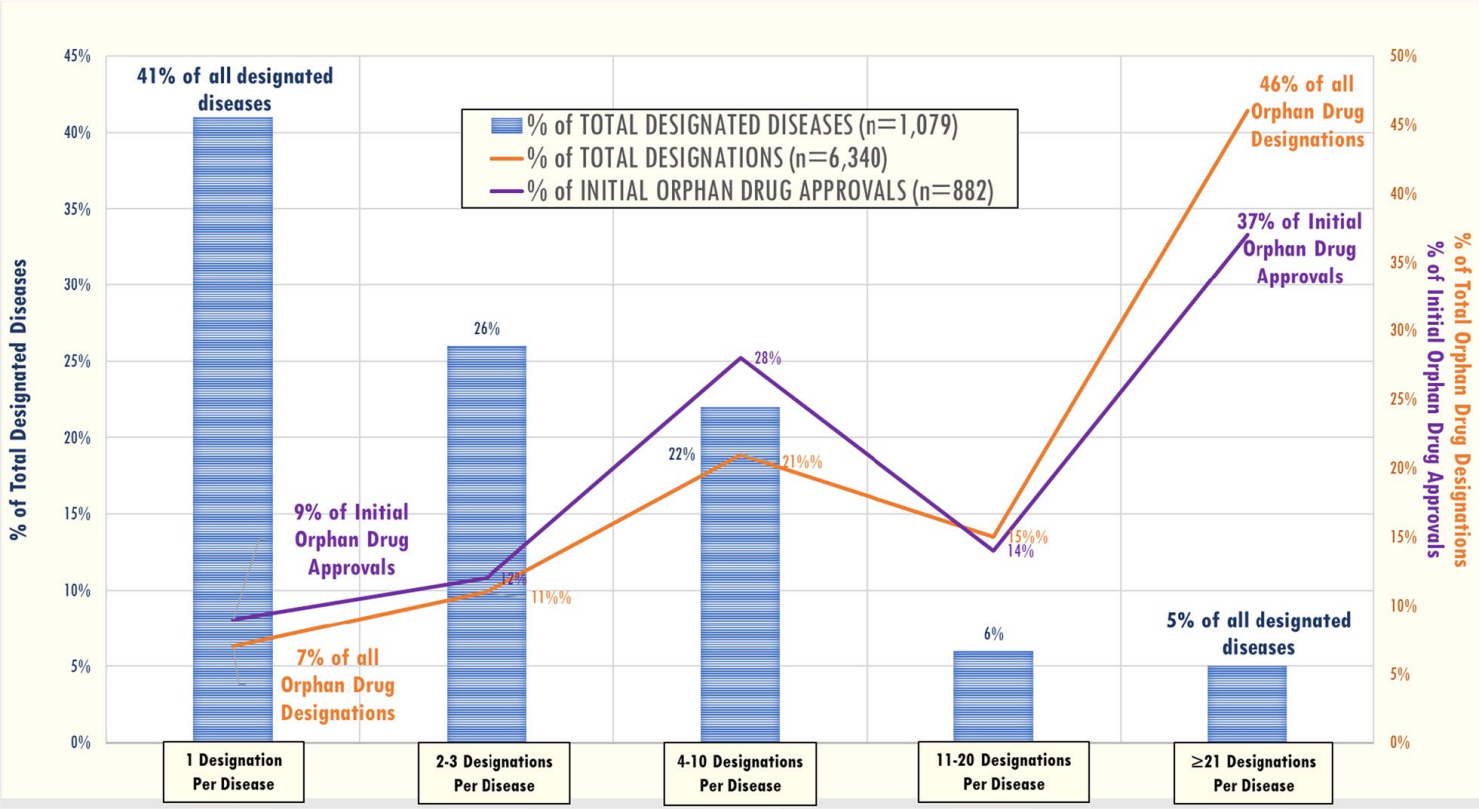
Few rare diseases account for a large percentage of orphan drug designations and initial orphan drug approvals, 1983–2022

Diseases with between one and three designations per disease represented two-thirds of all the designated diseases but comprised only 18% of designations and 21% of initial orphan drug approvals, whereas diseases with 21 or more designations represented only 5% of all the designated diseases but accounted for 46% of the designations granted and 37% of initial orphan drug approvals (Fig. 2).

The most common therapeutic areas represented by orphan drug designations were: oncology (38%), neurology (14%), and infectious diseases (7%). (Table 1) Initial orphan drug approvals followed a similar therapeutic area pattern: oncology (38%), infectious diseases (10%), and neurology (10%).

**Table 1:** Orphan drug designations and initial orphan drug approvals, by therapeutic area, 1983–2022

Therapeutic area	Designations % (6340)	Initial orphan drug approvals % (882)
Oncology	38% (2405)	38% (333)
Neurology	14% (892)	10% (84)
Infectious Diseases	7% (461)	10% (90)
Metabolism	6% (370)	7% (61)
Hematology	5% (306)	8% (69)
Pulmonary	4% (280)	2% (19)
Gastroenterology	4% (243)	3% (25)
Transplant	4% (239)	2% (18)
Ophthalmology	3% (200)	2% (19)
Vascular	2% (155)	2% (21)
Rheumatology	2% (150)	3% (26)
Endocrinology	2% (147)	5% (43)
Dermatology	2% (103)	1% (8)
Pharmacology/Toxicology/Poisoning/Chelators	2% (99)	2% (22)
Nephrology/Uurology	1% (86)	2% (15)
Immunology	1% (76)	2% (14)
Cardiology	1% (50)	1% (8)
Orthopedics	1% (43)	< 1% (4)
Obstetrics and Gynecology	< 1% (19)	< 1% (2)
Otorhinolaryngology	< 1% (10)	< 1% (0)
Nutrition	< 1% (6)	< 1% (1)

The top 15 diseases accounted for 25% of all designations, and the top 68 diseases accounted for 50% of all designations.

Cancers represented the majority of diseases with the most designations and initial approvals: they accounted for nearly 60% (n = 14) (Table 2) of the top twenty-five most designated diseases and 60% (n = 15) of the diseases with most initial orphan drug approvals. (Table 3) The non-oncologic diseases with the most designations were: amyotrophic lateral sclerosis, cystic fibrosis, HIV, idiopathic pulmonary fibrosis, and sickle cell disease.

**Table 2:** Top 25 diseases with the most orphan drug designations, 1983–2022

Disease	Therapeutic area	Designations	Initial orphan drug approvals
Malignant pancreatic neoplasm	Oncology	185	4
Acute myeloid leukemia	Oncology	183	14
Multiple myeloma	Oncology	130	19
Glioma	Oncology	129	4
Metastatic melanoma	Oncology	120	16
Amyotrophic lateral sclerosis	Neurology	119	5
Cystic fibrosis	Pulmonary	108	8
HIV infectious disease*	Infectious Diseases	92	23
Ovarian cancer*	Oncology	91	8
Hepatocellular carcinoma	Oncology	89	11
Gastric cancer	Oncology	80	4
Glioblastoma	Oncology	78	0
Idiopathic pulmonary fibrosis	Pulmonary	76	2
Sickle cell disease	Hematology	68	5
Graft versus host disease	Transplant	63	3
Duchenne muscular dystrophy	Neurology	63	5
Pulmonary arterial hypertension	Vascular	58	9
B-cell chronic lymphocytic leukemia	Oncology	57	12
Soft tissue sarcoma	Oncology	56	6
Acute lymphoblastic leukemia	Oncology	55	8
Solid organ transplant rejection	Transplant	49	7
Myelodysplastic syndrome	Oncology	49	5
Huntington disease	Neurology	46	2
Small cell lung carcinoma	Oncology	43	5
Systemic sclerosis	Rheumatology	42	1

*No longer considered a rare disease by FDA as total disease prevalence is currently calculated to be greater than 200,000 affected people in the US

**Table 3:** Top 25 diseases with the most designations with at least one orphan drug approval, 1983–2022

Disease	Therapeutic area	Initial orphan drug approvals	Designations
HIV infectious disease*	Infectious Diseases	23	92
Multiple myeloma	Oncology	19	130
Non-small cell lung carcinoma	Oncology	18	35
Metastatic melanoma	Oncology	16	120
Acute myeloid leukemia	Oncology	14	183
B-cell chronic lymphocytic leukemia	Oncology	12	57
Hepatocellular carcinoma	Oncology	11	89
Follicular lymphoma	Oncology	11	37
Isolated congenital growth hormone deficiency	Endocrinology	10	23
Pulmonary arterial hypertension	Vascular	9	58
Hemophilia B	Hematology	9	23
Ovarian cancer*	Oncology	8	91
Diffuse large B-cell lymphoma	Oncology	8	33
Cystic fibrosis	Pulmonology	8	108
Chronic myelogenous leukemia	Oncology	8	38
Acute lymphoblastic leukemia	Oncology	8	55
Solid organ transplant rejection	Transplant	7	49
Thyroid cancer	Oncology	7	13
Lennox–Gastaut syndrome	Neurology	7	16
Soft tissue sarcoma	Oncology	6	56
Neuroendocrine neoplasm	Oncology	6	24
Malaria	Infectious Diseases	6	28
Mantle cell lymphoma	Oncology	6	23
Homozygous familial hypercholesterolemia	Metabolism	6	16
Immune thrombocytopenic purpura	Hematology	6	22

*No longer considered a rare disease by FDA as total disease prevalence is currently calculated to be greater than 200,000 affected people in the US

The non-oncologic diseases with the most initial orphan drug approvals were: HIV, growth hormone deficiency, pulmonary arterial hypertension, hemophilia B, and cystic fibrosis.

## Discussion

Clinicians have heard the adage, ‘when you hear hoof beats, think of horses, not zebras’ to remind them that when unsure of a diagnosis, common diseases are the most likely etiology. However, current estimates suggest that there are approximately 7000–10,000 rare diseases, which cumulatively affect more than 30 million Americans. This research sought to investigate how many of these rare diseases have an FDA-approved treatment or have been investigated in a drug development effort.

Given this 7000–10,000 estimate of the number of rare diseases, our results indicate that, over the past 40 years of the ODA, 4–6% of rare diseases have at least one marketing approval. Additionally, the result that 1,079 diseases have received orphan drug designation indicates that 11–15% of all rare diseases have at least one product that has been studied and shown some promise for use in diagnosing, preventing, or treating them.

Our results also suggest significant concentration in orphan drug designations and approvals: only 5% (n = 54) of orphan-designated diseases account for nearly 50% of all orphan drug designations and 37% of initial orphan drug approvals. Twenty diseases have had more than 50 designated drugs studied for them. These results are primarily driven by oncology products: 70% of the top ten most designated and approved rare diseases and nearly 40% of all orphan drug designations and initial approvals are drugs developed for rare cancers. This concentration is not surprising considering the scientific progress that has been made in the field over the last two decades, including advances in basic and translational research that have led to new molecular targets for drugs and a better understanding of those targets. This has been driven, in part, by significant federal investments in finding a cure, such as the initiation of the Cancer Moonshot in 2016 [13].

The expansion of drug development for rare cancers could conceivably be replicated in other diseases in the future. For example, there is hope that product development and resultant drug approvals could be on the horizon for rare neurodegenerative diseases such as amyotrophic lateral sclerosis (ALS) facilitated by recent legislation such as the Accelerating Access to Critical Therapies for ALS Act of 2021 [14], increased public awareness, and promising scientific advances.

In contrast to those heavily designated diseases, there are over 400 rare diseases with only one orphan drug designation. It is notable that despite having only “one shot on goal”, nearly 10% of these designations have eventually resulted in at least one FDA approval. The large number of single designation rare diseases indicates ongoing activity across a wide and growing spectrum of previously overlooked conditions and could be interpreted as a hopeful sign for patients diagnosed with these diseases [15].

Since the enactment of the ODA, there are numerous examples of rare diseases and conditions that were once considered to be untreatable and ultimately fatal that are now manageable as a result of one or more orphan drugs. One example is HIV, a disease that is no longer considered a “death sentence" because of the innovative antiviral therapies that were developed in the 1980s and 1990s, some of which received the financial incentives provided by the ODA. Similarly, development of orphan-designated drugs that target the basic defect in cystic fibrosis (CF)—the CF transmembrane conductance regulator (CFTR) ion channel—has resulted in significantly increased life expectancy for patients with this devastating disease [16].

However, these successes have not occurred in a vacuum. There has been significant policy discussion that orphan drugs now represent close to half of all new drugs being approved in recent years. A central concern is that this trend indicates that resource allocation, in the form of industry research and development spending, has gone toward rare diseases and away from more common diseases [17]. This is a significant public health issue, given the vast unmet need in many of these conditions, such as Alzheimer's disease, coronary artery disease, and systemic lupus erythematosus. However, given that only 15% of rare diseases and conditions have ever received a designation, and only about 5% have an orphan drug approved for them (which are not always disease-modifying nor curative), significantly more drug development, not less, will be needed if we wish to find treatments for all rare disease patients.

The recent volume of orphan designations demonstrates that there is a robust pipeline of promising products being developed for rare disease patients. Development of novel gene and cell therapies, antisense oligonucleotides, and innovative targeted small molecule drugs among other groundbreaking therapeutic technologies have been designated and are currently being studied in human subjects with the financial support provided by the ODA’s incentives. There is hope that the success of these innovative platforms could lead to approvals for a wide range of rare diseases for which these products could be applicable.

Additionally, the publicly-available list of orphan-designated drugs may eventually benefit researchers of related rare disorders that share similar pathophysiology who are interested in exploring promising drugs to inform repurposing efforts. In the most recent decade, the number of orphan drug designations has more than doubled over the previous decade, and over 3000 unique drugs and biologics have received orphan drug designation since the initiation of the ODA [18, 19]. The fact that the number of designations is currently lengthy and increasing further suggests that potential targets for repurposing could be identified and investigated to find more rare disease therapeutics in future years.

The success of the ODA has inspired similar legislative initiatives in other global organizations including the European Union Orphan Medicinal Products Regulation, Health Canada Orphan Drug Policy, and Orphan Drug Acts in Japan, Australia, and South Korea. These descendants of the ODA also provide incentives for the development of drugs for rare diseases such as fee reductions, research and development tax incentives, and market exclusivity [20–22]. Future research could utilize the methodology of this study to investigate the number of rare diseases with approved treatments in other regulatory environments.

This study should be considered a first step in understanding the complete landscape of drug development and therapeutic options for rare disease patients. Despite being a remarkably heterogeneous group of disorders, it is not uncommon for rare diseases to be grouped together and treated as a monolith and therefore, this analysis deliberately focused on individual rare diseases and conditions. Using this more granular view of rare diseases provides an opportunity to explore other potential areas of future research including evaluating the correlation between disease prevalence and the number of orphan drug designations and approvals (to determine whether relatively higher disease prevalence is associated with greater numbers of development programs and approvals; [23]) and the proportion of rare disease patients who benefit from approved therapies (thereby calculating the tangible impact of these products on unmet need).

Finally, future research should also investigate the question of how many rare diseases have therapeutic options with a more holistic view of “treatment”. The current research has focused specifically on drugs and biologics, however, other methods of treatment, such as surgical or medical devices are also utilized in rare diseases. For example, rare diseases like congenital cholesteatoma or gastroschisis are primarily corrected surgically, and therefore diseases like these are not incorporated into current estimates of the number of rare diseases with treatment options. Research that employed a wider definition of treatment could provide a more comprehensive picture of the clinical landscape for all rare diseases.

### Limitations

The primary limitation of this study is that using orphan drug designations as a proxy for the entirety of rare disease drug development may not be complete. A drug developer may request orphan-drug designation at any time prior to submitting an application for marketing approval to the FDA for that drug for that disease. Therefore, it is possible that some companies do not apply when in the preclinical phase and end the program before applying. This may be especially true for sponsors that are nonprofits (e.g., patient groups or academics), for whom the early-stage financial incentives (e.g., tax credits) are not applicable. Additionally, the data does not capture drugs that have been studied for a rare disease but have no plans for commercialization (e.g., purely academic studies).

We also acknowledge that categorizing certain designations into diseases is inherently subjective and the categorization process can be inconsistent. We have minimized this bias through the use of a validated ontology resource, as well as an iterative review process performed by both authors.

Finally, not all products approved for rare diseases receive orphan designation prior to approval. While there are very limited instances of this, it is possible that we did not capture all approvals for rare diseases. There are also approvals that occurred before the ODA was enacted, which we were also unable to capture.

## Conclusion

When the ODA was created, its goal was to incentivize the development of drugs for rare diseases and conditions. It seems unlikely that the ODA’s advocates in the early 1980s could have imagined the growth and scale of the rare disease drug development landscape and ecosystem in the present day. After four decades of its grants, designations, incentives, and ensuing orphan drug approvals, it is clear that the ODA is a transformative piece of public health legislation that has served to better characterize, manage, and amplify the zebra hoof beats of rare diseases.

## Data Availability

The dataset analyzed for this research may be available upon request to the authors. The underlying data used to generate the dataset is publicly available via the website https://www.accessdata.fda.gov/scripts/opdlisting/oopd/.

## Abbreviations

ALS: Amyotrophic lateral sclerosis
CF: Cystic fibrosis
FDA: Food and Drug Administration
HIV: Human immunodeficiency virus
ODA: Orphan Drug Act
